# A scenario analysis-based optimal management of water resources supply and demand balance: A case study of Chengdu, China

**DOI:** 10.1371/journal.pone.0267920

**Published:** 2022-05-16

**Authors:** Yang Yu, Tianyu Zhou, Rui Zhao, Zhanglong Li, Chao Shen

**Affiliations:** 1 Faculty of Geosciences and Environmental Engineering, Southwest Jiaotong University, Chengdu, Sichuan Province, China; 2 Chengdu Engineering Corporation Limited, Power China, Chengdu, Sichuan Province, China; 3 Urban Water Environment Treatment Engineering Technology Research Center of Sichuan Province, Chengdu, Sichuan Province, China; Shenzhen University, CHINA

## Abstract

Water resources scarcity has threatened the coordinative development of demographics, society and economy. As a typical rapidly urbanizing area and an emerging megacity in China, Chengdu is confronting the pressure of inadequate water supply. The present study divides the macroeconomic factors that affect the water resource supply and demand balance into six major subsystems: water resources supply, water demand, water drainage, population, ecological environment and economy. The combining variable interaction description and predictive simulation models are applied to simulate the water supply and demand ratio (S:D) from 2005 to 2035. Further, this study designs different development scenarios to simulate the change of S:D ratios by altering the parameter values of driving factors. The results show that: (1) the S:D ratio will decline if the current development scenario continues, implying the serious water resources shortage and the severe water supply-demand conflict in Chengdu; (2) socio-economic water demand and wastewater/rainwater reuse are the key driving parameters of S:D ratio, especially the water consumption per ten thousand yuan of industrial value-added; (3) the S:D ratio will increase from 0.92 in the current baseline scenario to 1.06 in the integrated optimization scenario in 2025, and the long-term planning brings 2035 from 0.71 to 1.03, with the proportion of unconventional water supply rise to 38% and 61%, respectively. This study can provide a decision-making tool for policy-makers to explore plausible policy scenarios necessary for bridging the gap between the water supply and demand in megacities.

## Introduction

With rapid urbanization, the conflicts between the water supply and water demand in megacities are becoming even more serious, which has been a long-term challenge for urban sustainable development [1, 2]. Water supply is involved with several factors, such as population growth, economic urbanization, land utilization, agricultural production, wastewater recycling, etc. [3]. The nexus among society, the environment, and water systems has intensified the shortage of water supply [4, 5]. To improve the water utilization efficiency, it is necessary to identify the key factors that drive the conflicts, as well as to reveal complex interactions between the factors to solve allocation in multiple systems rationally [6, 7].

System dynamics, because of their capability of simulating a multivariable, multi-loop and highly nonlinear evolution process [8], can be embodied into the simulation for sustainable utilization of water resources [9, 10]. The application of SD in water resources management has transformed from predictive simulation to variable interaction description [11, 12]. The former describes the future spatiotemporal distribution of the available water resources, and the sustainability of water supply and demand influenced by human activities and climate change [13]. For example, Cheng et al. [14] adopted the system dynamics to simulate the variation of the water resources carrying capacity in Suzhou city, China. Hoekema and Sridhar [15] proposed a system dynamics-based river planning model to simulate the variation of surface water supply in terms of climate change impacts. Similarly, Ganji and Nasseri [16] applied the system dynamics approach to simulate the impact of climate change on agricultural production. Sahin et al. [17] presented a system dynamic model by integrating desalination into the water supply network, to explore the optimal water distribution schemes. To investigate the effects of climate change on both the quality/quantity of the water resources system, Duran-Encalada et al. [18] developed an SD model to simulate policies and decisions that have the potential to improve temperature/precipitation conditions and prevent water quality/quantity damages. Bagheri and Babaeian [19] adopted an SD model to analyze policies to improve water security in terms of system vulnerability. However, the above studies have not taken the interaction between socio-economic activities and water resources supply into account.

The variable interaction description fills the gap, which contributes to focusing on the interaction among water supply and socio-economic development, to identify the key factors that influence the balance of water supply and water demand [20]. Sun et al. [1] built a system dynamics model that is composed of five subsystems: economy, population, water supply and demand, land resources, and water pollution, to obtain an optimal program between water distribution and socio-economic development. Li et al. [21] developed an SD model to describe the water resources vulnerability, which is affected by the water resources system and socio-economic system. Gozini et al. [22] proposed a system dynamic model to investigate the water-energy nexus under various incentive policies. Legal, economic, technical and necessary administrative measures can coordinate regional water demand and available water supply effectively [3, 23, 24]. However, current water resources simulation or evaluation was usually carried out under baseline scenario and rarely coupled the predictive simulation model with the variable interaction description model, ignoring the important driving sensitive factors for water resource supply and demand system simulation. In addition, existing studies have not considered the impact of external policy implementation on water use structure allocation. Furthermore, most of the studies are based on national or watershed scales without integrating local policies and planning, resulting in poor implementation of research results and insufficient practical guidance.

The Chengdu city has been entrusted with the important mission of building a national central city in China. The development of Chengdu city has confronted challenges related to water shortage. This study developed a water balance system for Chengdu city, which is constituted by six subsystems, including water supply, water demand, economy, population, ecology, and water drainage. The combining variable interaction description and predictive simulation models are applied to analyze the water cycle, and then the driving factors of water supply and water demand are identified. According to the blueprint of Chengdu City (2020–2035), the optimal scheme of water supply and demand balance is proposed to provide policy implications on urban sustainable utilization of water resources.

## Materials and methods

### Study area

Chengdu city is located in the western part of Sichuan Basin, China. The Chengdu Plain is an alluvial fan plain formed by the Minjiang River and Tuojiang River, and the two rivers flow through the city. The location is at longitude 102°54′-104°53′ E and latitude 30°05′-31°26′ N. It covers an area of 14,335 km^2^ and the length of east-west and north-south are 192 km and 166 km, respectively. The climate in Chengdu is a subtropical monsoon climate. The annual average temperature is 16°C and annual rainfall ranges from 873–1265 mm. The water supply for socio-economic water use in Chengdu is dominated by Minjiang transit water. In 2018, the total population of Chengdu city was 14.76 million, the gross domestic product (GDP) was 1534.28 billion RMB (236.82 billion USD), and the GDP per capita was 103,900 RMB (16,600 USD).

Since the 1980s, water resources scarcity has become the prominent challenge for restricting sustainable development in Chengdu city [25]. The water demand increases greatly due to the rapid growth of the population and economy. The water resource per capita is only 696 m^3^, which is accounted for one-fourth of the national average. When Chengdu city aims to the development of the national central city, the city will continue further expansion which may aggravate the pressure of water supply.

### Data sources

The historic socio-economic data from 2005 to 2018 are derived from *the Chengdu Statistical Yearbook* [26], *Sichuan Statistical Yearbook* [27], and *the China Urban Construction Statistical Yearbook* [28], including registered and non-registered population growth rate, green land area per capita, etc. Water resources, water consumption, and water utilization rate of various sectors are derived from *the Water Quota of Sichuan Province* [29] and *Chengdu Water Resources Bulletin* [30], including domestic water demand per capita, water consumption per unit green area, etc. Moreover, the parameters used in the model prediction (2018–2035), except for the initial values in 2018, others are set according to local policies and planning reports, including *the 13th Five-Year Plan of Chengdu Forestry and Garden Development* [31], *Urban Green Space System Planning of Chengdu (2019–2035)* [32], and *the 13th Five-Year Plan of Chengdu Water Development* [33].

### SD model construction

This study mainly concentrates on a system for unveiling the balance between water resource supply and demand in megacities, which considers the interaction between socio-economic activities and water resources supply. Furthermore, the above two factors are classified into six subsystems to simulate the changes in water supply and demand ratios. The combining variable interaction description and predictive simulation models are implied to provide optimal schemes of water supply and demand balance. These optimal schemes proposed by scenario analysis can lay the foundation for the sustainable planning and management of urban water resources.

**Fig 1:**
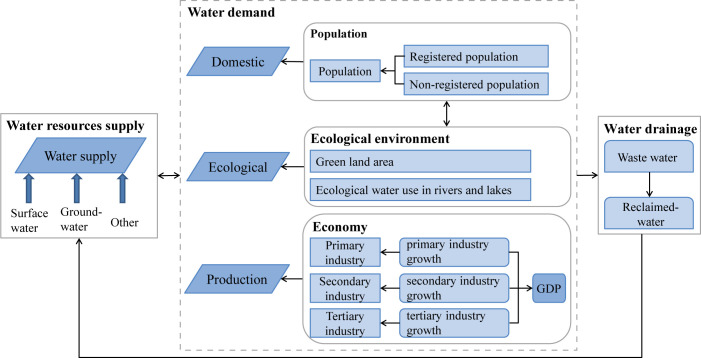
Diagram of the water cycle in each subsystem.

The administrative boundary of Chengdu city is used as the spatial boundary of the SD model. The water resources supply-demand balance system is mainly composed of six subsystems: water resources supply, water demand, water drainage, population, ecological environment and economy. The water resources circulation relationship among these subsystems is shown in Fig 1.

#### Causal-loop relationship

**Fig 2:**
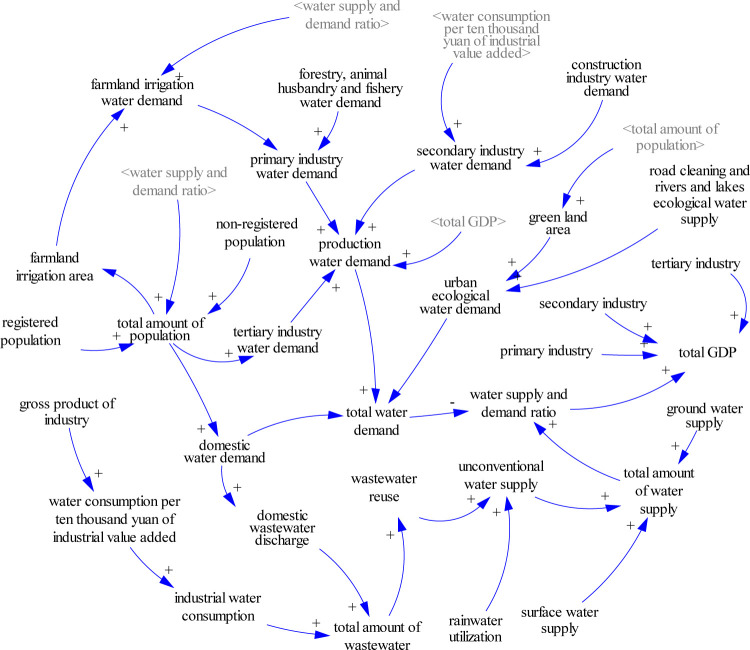
The causal-loop diagram of the Chengdu water cycle system.

The SD model has a total of 55 variables and constants (as shown in the S1 Table), including 8 state variables, 8 rate variables, 32 auxiliary variables, and 7 constants. Their equations among the above variables are given in the S2 Table. The causal loop system is shown in Fig 2. A positive link is denoted with a “+” at the end of the arrow, which represents two variables changing in the same direction. And a negative link is denoted with a “-”, this means the opposite. There are two main causal loops in the SD model:

Reinforcing loop:

1. Total amount of population→domestic water demand→total water demand→water supply and demand ratio→total amount of population;
2. Total GDP→production water demand→total water demand→water supply and demand ratio→total GDP;
3. Industrial wastewater discharge→total amount of wastewater→wastewater reuse→total amount of water supply→water supply and demand ratio→total GDP→industrial water consumption→industrial wastewater discharge;
4. Domestic wastewater discharge→total amount of wastewater→wastewater reuse→total amount of water supply→water supply and demand ratio→total amount of population→domestic water demand→domestic wastewater discharge;

Balancing loop:

1. Green land area→urban ecological water demand→total water demand→water supply and demand ratio→total amount of population→green land area;
2. Farmland irrigation water demand→primary industry water demand→production water demand→total water demand→water supply and demand ratio→farmland irrigation water demand;
3. Industrial water consumption→secondary industry water demand→production water demand→total water demand→water supply and demand ratio→gross product of industry→industrial water consumption.

**Fig 3:**
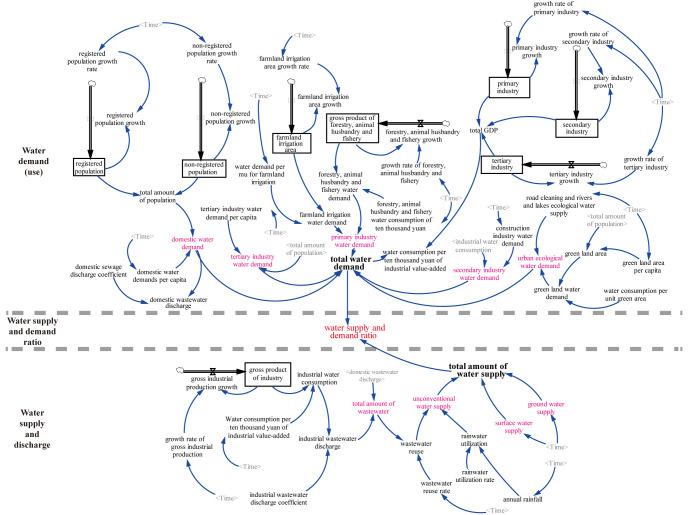
Diagram of Chengdu water resources supply and demand system.

Fig 3 shows the systemic interaction relationship among these six subsystems, in which the water supply depends on surface water, groundwater and unconventional water resources, such as rainwater and reclaimed water. The wastewater reuse acts as input to the unconventional water resources with a time delay of one month, as it cannot be used as input until effective treatment [34]. Water demand includes industry, agriculture, urban ecology and domestic use. The S: D ratio was used to describe the water resources cycle, which is further used to reflect the water resource variations.

#### Input parameters

Three approaches have been selected to obtain the input parameters, including the regression analysis [35], the multi-year average of measured value [36], and the variable value keeps constant with the latest year [24, 37]. The input parameters and their associated values are listed in Table 1.

**Table 1:** Interpretation of the key parameters.

Parameter	Initial value	Interpretation
Registered population growth rate	1.2%	The growth rate can be calculated according to observed data in the *Chengdu Statistical Yearbook* [26] and *Sichuan Statistical Yearbook* [27].
Non-registered population growth rate	-1.11%	
Domestic water demand per capita	78.65 m^3^/(capita_*_year)	The value is derived from the *Water Quota of Sichuan Province* [29] and the *Chengdu Water Resources Bulletin* [30].
Water consumption per unit green area	0.91 m^3^/(m^2^_*_year)	This unit water consumption is derived from *the Water Quota of Sichuan Province* [29].
Green land area per capita	15 m^2^/capita	The green land area per capita can be measured by total green land area divided by the total amount of population, in which the total green land area is referenced from the China Urban Construction Statistical Yearbook [28], *The 13th Five-Year Plan of Chengdu Forestry and Garden Development* [31] and *Urban Green Space System Planning of Chengdu* (2019–2035) [32].
Water demand per mu for farmland irrigation	515 m^3^/(mu_*_year)	The value is derived from *the Water Quota of Sichuan Province* [29] and *the Chengdu Water Resources Bulletin* [30].
Wastewater reuse rate	0.36	The wastewater reuse rate is referenced from *the 13th Five-Year Plan of Chengdu Water Development* [33], its values in 2015 and 2020 are 0.1 and 0.4, respectively.
Domestic sewage discharge coefficient	0.85	This coefficient is referred to from similar studies and field investigations.
Industrial wastewater discharge coefficient	0.30	This coefficient is referred to from similar studies and field investigations.

#### Validity analysis of extracted data

Before the data which is extracted from statistics yearbooks and bulletins were used for SD model testing and verification, validity analysis was conducted on the extract values of the major variables to reduce and mitigate the possible subjective influence [38]. Validity in this context refers to the degree of consistency of data used in this study. Therefore, the Cronbach Alpha coefficient was used as an assessment indicator to measure the internal consistency of our standardized data [39, 40]. If the coefficient is greater than 0.9, the extracted data set shows high validity, while a value between 0.9 and 0.7 is also acceptable. However, if the coefficient drops to between 0.7 and 0.5, then some data need to be revised, and some may need to be abandoned if the value falls below 0.5 [38]. The Cronbach Alpha coefficient can be calculated as:

$$\alpha =(\frac{n}{n-1})*(1-\frac{\sum_{i=1}^{n}\sigma_{i}^{2}}{\sigma_{S}^{2}})$$ (1)

where α is the validity coefficient and *n* denotes the number of variables, while $\sigma_{i}^{2}$ represents the variance of the *i*^*th*^ variable during the assessment period, $\sigma_{S}^{2}$ is the variance of the sum of the variables among assessment period.

#### Model validation

The relative error between simulated and observed values is adopted to evaluate the applicability of the developed SD model. It can further demonstrate the deviation between the simulated value and the observed value, as shown in Eq (2).

$$R=\left|\frac{S_{i}-M_{i}}{M_{i}}\right|$$ (2)

where *S*_*i*_ and *M*_*i*_ are the simulated and measured data of variable *i*, respectively.

The actual statistical data from 2005 to 2015 were taken as the test sample set, and the SD model was applied to forecast the value of major variables in 2016–2017. The results showed that relative errors of the main variables, such as the gross product of primary industry, population, domestic, farmland irrigational and ecological water demand, industrial water consumption, and secondary industry water demand were all less than 10%, shown in Table 2. The model is considered reasonable for further scenario analysis [1, 41].

**Table 2:** Relative errors of the main variables between simulated data and measured data.

Variables	2016			2017			2018		
	Measured data	Simulated data	Relative error (%)	Measured data	Simulated data	Relative error (%)	Measured data	Simulated data	Relative error (%)
The gross product of primary industry (10^8^ CNY)	475	458.29	3.52	501	494.98	1.20	523	533.71	2.05
The gross product of secondary industry (10^8^ CNY)	5202	5413.90	4.07	5998	6216.27	3.64	6516	7105.12	9.04
The gross product of tertiary industry (10^8^ CNY)	6493	6537.22	0.68	7390	7589.34	2.70	8304	8785.02	5.79
The gross product of industry (10^8^ CNY)	4479	4545.20	1.48	5217	5243.86	0.51	5664	6023.22	6.34
Registered population (10^4^ people)	1232	1231.88	0.01	1247	1246.66	0.03	1261	1261.62	0.05
Non-registered population (10^4^ people)	234	233.90	0.04	231	231.30	0.13	229	228.73	0.12
Domestic water demand (10^8^ m^3^)	9.66	9.65	0.10	9.92	9.92	0.00	15.09	15.08	0.07
Industrial water consumption (10^8^ m^3^)	12.14	11.47	5.52	12.78	12.08	5.48	9.23	9.81	6.28
Farmland irrigation water demand (10^8^ m^3^)	27.56	28.58	3.70	28.11	28.1	0.04	29.66	29.66	0.00
Primary industry water demand (10^8^ m^3^)	32.34	33.36	3.15	32.91	32.9	0.03	30.8	31.3	1.62
Secondary industry water demand (10^8^ m^3^)	13.98	13.47	3.65	14.67	14.88	1.43	11.42	12.39	8.49
Tertiary industry demand (10^8^ m^3^)	3.38	3.31	2.07	3.30	3.27	0.91	3.51	3.64	3.70
Urban ecological water demand (10^8^ m^3^)	1.67	1.72	2.99	1.69	1.71	1.18	1.18	1.21	2.54

## Simulation analysis and results

### Validity analysis

We implied the validity analysis on extracted original data encompassing the period between 2008 and 2018 for Chengdu, the standardized data using the min-max normalization method is shown in Table 3. We then obtained the Cronbach Alpha coefficient with 0.83; the results show that all extracted data are credible because its validity coefficient is greater than 0.7.

**Table 3:** Variable standardization values for Chengdu between 2008 and 2018.

Variables	The gross product of primary industry (10^8^ CNY)	The gross product of secondary industry (10^8^ CNY)	The gross product of tertiary industry (10^8^ CNY)	The gross product of industry (10^8^ CNY)	Registered population (10^4^ people)	Non-registered population (10^4^ people)	Domestic water demand (10^8^ m^3^)	Industrial water consumption (10^8^ m^3^)	Farmland irrigation water demand (10^8^ m^3^)	Primary industry water demand (10^8^ m^3^)	Secondary industry water demand (10^8^ m^3^)	Tertiary industry demand (10^8^ m^3^)	Urban ecological water demand (10^8^ m^3^)
2008	0.9914	1.0000	1.0000	1.0000	1.0000	0.8689	1.0000	0.0000	0.9338	0.0431	0.7270	1.0000	0.9019
2009	1.0000	0.9606	0.9355	0.9557	0.8923	1.0000	0.9769	0.5908	0.9380	0.0000	0.3124	0.9264	0.9019
2010	0.9329	0.8587	0.8504	0.8606	0.8230	0.0000	0.7967	0.2588	0.8097	0.4753	0.3956	0.8127	0.3126
2011	0.7675	0.7176	0.7434	0.7296	0.7186	0.1984	0.8703	0.1451	1.0000	0.8707	0.3182	0.5351	0.1082
2012	0.6859	0.5853	0.6593	0.6061	0.6448	0.1861	0.8176	0.1749	0.8191	0.6172	1.0000	0.7559	0.0000
2013	0.6659	0.4968	0.5747	0.5188	0.5373	0.2296	0.6901	0.6220	0.3964	0.5836	0.4482	0.5050	1.0000
2014	0.6506	0.4272	0.4797	0.4322	0.4299	0.2732	0.6659	1.0000	0.5846	0.7424	0.8978	0.0000	0.9716
2015	0.5875	0.3814	0.4006	0.3842	0.3224	0.3167	0.6407	0.3108	0.6172	1.0000	0.2511	0.4548	0.9615
2016	0.1882	0.2796	0.2791	0.2832	0.2149	0.3603	0.5967	0.2214	0.2208	0.3007	0.1007	0.3311	0.9487
2017	0.0863	0.1102	0.1408	0.1068	0.1075	0.4038	0.5681	0.0829	0.1630	0.2408	0.0000	0.3579	0.9468
2018	0.0000	0.0000	0.0000	0.0000	0.0000	0.4474	0.0000	0.8468	0.0000	0.4627	0.4745	0.2876	0.9936

### Supply: Demand ratio simulation

**Fig 4:**
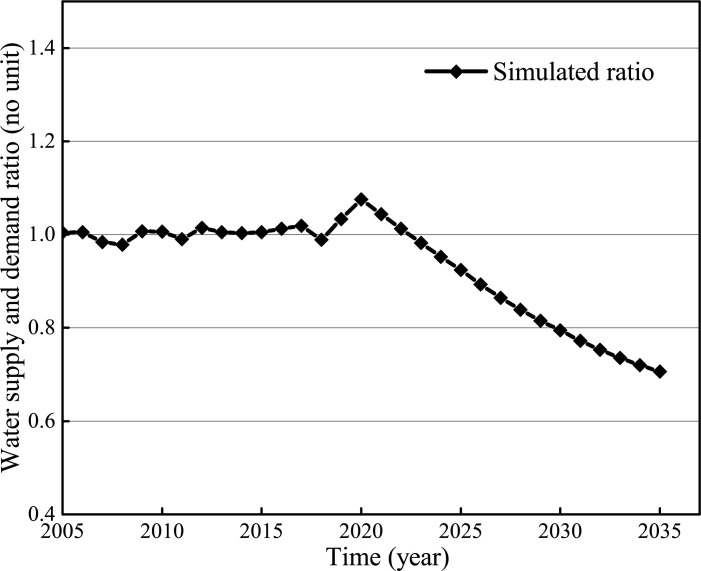
The simulated S:D ratio from 2005 to 2035.

The S: D ratio simulation is given in Fig 4. From 2005 to 2020, the ratios fluctuate around 1, with a slight increase, which indicates that water supply may satisfy the water demand, but such balance is fragile. After 2020, the pressure on water supply is overloaded due to the rapid economic and population growth, and the ratio begins to decline, from 0.92 in 2025 to 0.71 in 2035, implying a serious water shortage. In addition, the strictest water resources management in Chengdu and the *13th Five-Year Plan of Chengdu Water Development* [33] reported that the total water consumption in Chengdu would be confined to 6.93 billion m^3^ from 2020 and 7.1 billion m^3^ from 2030. Therefore, some measures must be taken to ensure the sustainable utilization of water resources in megacities.

### Driving factors identification

**Fig 5:**
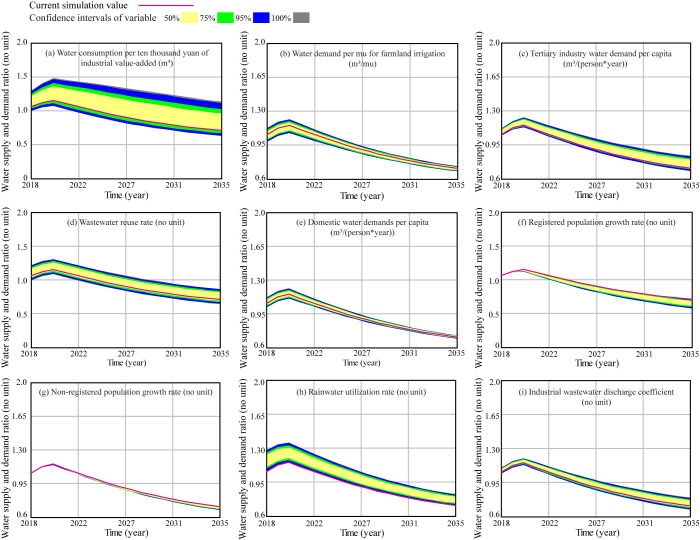
Influence of nine key variables on the water supply and demand ratio (current simulation value).

The imbalance between water supply and demand is incurred by the decrease in water supply and the increase in water demand caused by human activities [21]. Balancing water demand and supply conflicts among different water use structures with limited water resources is an indispensable measure to adapt to regional sustainable development [42]. Previous research has pointed out that the proportion of water consumption in farmland irrigation, industry production, tertiary industry, and daily living is the largest in China’s megacities [12, 43]. In addition, enhancing unconventional water resources utilization has also become an important breakthrough in resolving the contradiction [24]. Consequently, nine variables are selected to further identify the driving factors upon the S:D ratio, to lay the foundation for further scenario analysis (Table 4). The numerical values related to these parameters were determined randomly within their allowable range through 200 times iteration, and other parameters remained constant. In Fig 5, different colors represent different sensitive intervals. Yellow, green, blue, and gray color belts represent the simulated S:D ratios when the parameter belongs to the confidence intervals of 50%, 75%, 95% and 100%, respectively [24, 44].

**Table 4:** Parameters used for driving factors identification.

Parameters	Initial value	Test interval of the parameter
Water consumption per ten thousand yuan of industrial value-added (m^3^)	22.3	[0, 30]
Water demand per mu for farmland irrigation (m^3^/mu)	515	[450, 600]
Tertiary industry water demand per capita (m^3^/(person_*_year))	26.33	[0, 30]
Wastewater reuse rate (no unit)	0.3	(0, 1)
Domestic water demands per capita (m^3^/(person*year))	78.65	[50, 100]
Registered population growth rate (no unit)	0.012	[0.01, 0.1]
Non-registered population growth rate (no unit)	-0.011	[-0.01, 0.1]
Rainwater utilization rate (no unit)	0	(0, 10%)
Industrial wastewater discharge coefficient (no unit)	0.3	(0, 1)

The water consumption per ten thousand yuan of industrial value-added is the most sensitive to the simulated S:D ratio, especially when the water consumption is set within 50% of the confidence range, shown in Fig 5(A). It is further inferred that the water consumption per ten thousand yuan of industrial value-added within the range of [7.5, 22.5] may have a significant impact on the S:D ratio, by which the S:D ratio in 2035 will increase by 60.73%.

The S:D ratio is sensitive to the water demand per mu for farmland irrigation, shown in Fig 5(B), and the sensitive interval of 50% change range is [487.5, 562.5], the sensitive intervals for 75% and 95% are [468.75, 487.5] and [562.5, 581.25], and [453.75, 468.75] and [581.25, 596.25], respectively. If the water demand per mu for farmland irrigation is set within [453.75, 515], the S:D ratio is improved especially before 2032 as its increment rate will be higher than 10%.

The tertiary industry water demand per capita has a significant impact on S:D ratio, by which it will increase 19.51% in 2035, shown in Fig 5(C). Wastewater reuse rate shows a similar impact on the S:D ratio, when increasing from 0.3 to 0.75, the S:D ratio will increase from 0.71 to 0.82, increased by 22.38% in 2035, shown in Fig 5(D). When the value of domestic water demand per capita ranges between [56.25, 87.5], the S:D ratio will vary between [0.70, 0.73], increased by 3.01% in 2035, shown in Fig 5(E).

As can be seen from Fig 5(F) and 5(G), control of population growth before 2020 is conducive to the increase of the S: D ratio, while the contribution to the ratio in 2020–2035 is negligible. It is obvious that the S: D ratio is more sensitive to registered population growth rate than to non-registered population growth rate.

The increase of the rainfall utilization rate contributes to the increase of the S: D ratio, shown in Fig 5(H), which increased by 17.1% in 2035. The industrial wastewater discharge coefficient has an obvious influence on the S:D ratio, shown in Fig 5(I), which will increase from 0.71 to 0.77.

Based on the above analysis, 6 parameters are identified to have a significant impact on the S:D ratio, by which they give rise to at least 10% of the growth in 2035, i.e., water consumption per ten thousand yuan of industrial value-added, water demand per mu for farmland irrigation, tertiary industry water demand per capita, wastewater reuse rate, domestic water demand per capita and rainwater utilization rate. These parameters are selected for the following scenario analysis.

### Scenario analysis-based optimal scheme design

The above simulation results imply that Chengdu will endure a serious water shortage in the following years. Policy measures are thus undertaken to bridge the gap between water supply and demand [24]. According to *The standard of water quantity for city’s residential use* (GB/T 50331) [45] and *The instruction for domestic waste generation and discharge coefficient* [46] for water-saving cities issued by the Ministry of Housing and Urban-Rural Development and Ministry of Ecology and Environment of the People’s Republic of China, domestic water consumption per person L/(person_*_day) is allocated less than 72.3 m^3^/(person_*_year). In such context, the water consumption of the tertiary industry in Chengdu is below 7.7 m^3^/(person_*_year). Two scenarios are set as follows:

Scenario I: The domestic water consumption per capita is 90% of the planned water quota, that is, 65.1 m^3^/(person_*_year), and the tertiary industry water consumption per capita is reduced by 60% compared with the baseline value, that is, 15.7 m^3^/(person_*_year).

Scenario II: The domestic water consumption per capita is 75% of the planned water quota, that is, 54.2 m^3^/ (person_*_year), and the tertiary industry water consumption per capita is set as 7.7 m^3^/ (person_*_year).

**Fig 6:**
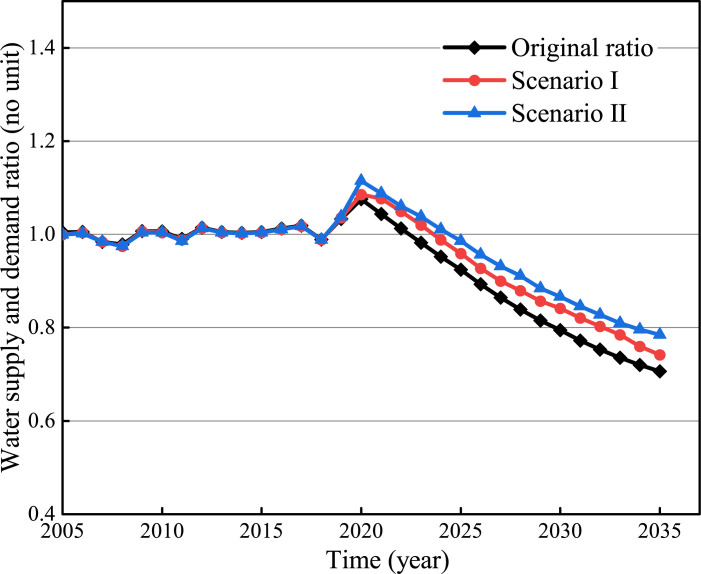
Optimization simulation results of household water conservation.

Fig 6 shows that water-saving can effectively improve the ratio of water supply and demand. In Scenario 1, the water consumption of domestic and tertiary industries in 2025 can be saved by 27% and 24% respectively. They will be further improved in 2035 by 33% and 28%, respectively. In Scenario 2, the water consumption of domestic and tertiary industries in 2025 can be reduced by 31% and 33%, respectively. They will be further improved in 2035 by 38% and 41%, respectively.

*Sichuan Water Saving Action Implementation Plan* [47] pointed out that water consumption per ten thousand yuan of industrial value-added should decrease by 23% and 28% in 2020 and in 2022, respectively, compared with that in 2015. In addition to decreasing water consumption in residential life and tertiary industry, there are two scenarios set based upon the water consumption per ten thousand yuan of industrial value-added:

Scenario III: The water consumption per ten thousand yuan of industrial value-added is 22.25 m^3^.

Scenario Ⅳ: The water consumption per ten thousand yuan of industrial value-added is 20.81 m^3^.

**Fig 7:**
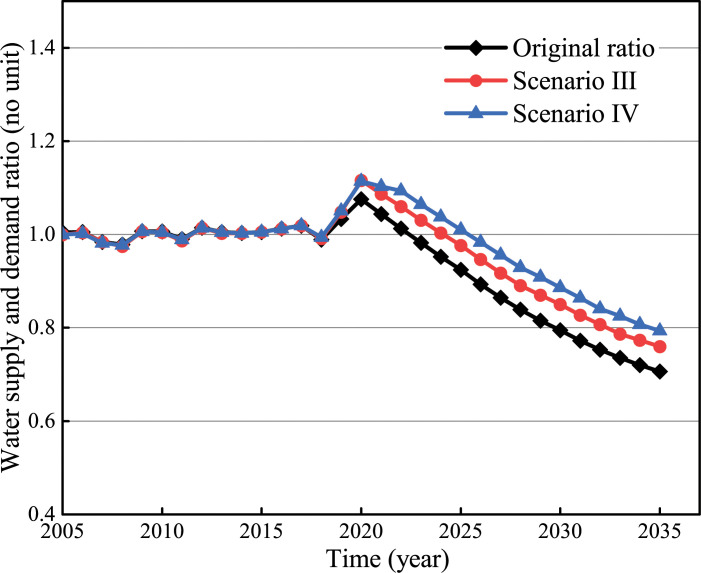
Optimization simulation results of industrial water conservation.

The S:D ratio is shown in Fig 7. The ratio will increase from 0.92 to 0.98 (Scenario III) and 1.01 (Scenario Ⅳ), respectively in 2025. Industrial water consumption can be saved by 18.18% and 29.12%, respectively. The ratio will increase from 0.71 to 0.76 (Scenario III) and 0.79 (Scenario Ⅳ) in 2035. In this circumstance, industrial water consumption can be saved by 18.18% and 29.10%, respectively.

Farmland irrigation accounts for 50% of the total water consumption, and 77.58% of the primary industry water demand in Chengdu. Improvement in the efficiency of agricultural water use is essential to control total water consumption [48]. Outline of *the 13th Five-year plan of Chengdu water development* [33] stated that the coefficient of farmland irrigation should increase to 0.56 in 2020 and 0.6 in 2030, respectively. Thus, two scenarios are set as follows:

Scenario V: The Water demand per mu for farmland irrigation is set as 505 m^3^/mu.

Scenario VI: The Water demand per mu for farmland irrigation is set as 485 m^3^/mu.

**Fig 8:**
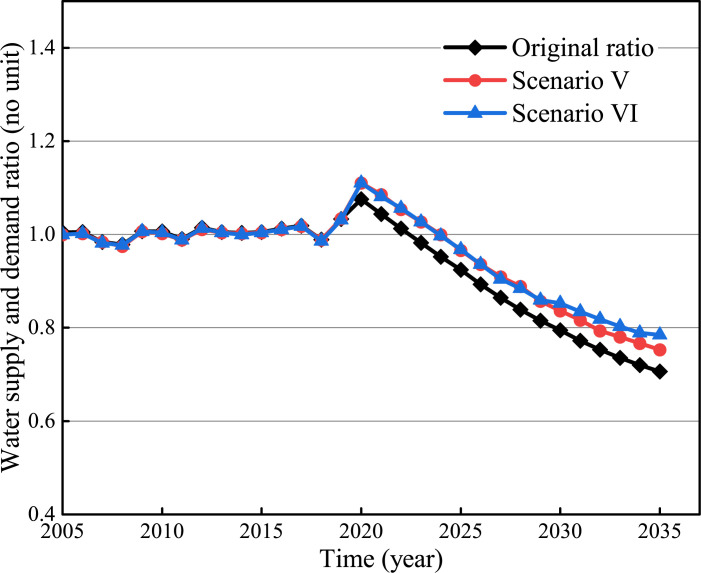
Optimization simulation results of agricultural water conservation.

The S: D ratio simulation results are shown in Fig 8. Agricultural water conservation can significantly increase the S: D ratio, increasing from 0.71 to 0.75 (Scenario V) and 0.79 (Scenario VI) in 2035, respectively. In addition, the agricultural water demand can be saved by 4% and 19% in 2035, respectively.

According to *The 13th Five-Year Plan of Chengdu Water Development* [33], the wastewater reuse rate in Chengdu city should be more than 40% by 2020 and 50% by 2025. Consequently, two scenarios are set as follows:

Scenario VII: The wastewater reuse rate is 0.4 and the rainwater utilization rate is 1%.

Scenario VIII: The wastewater reuse rate is 0.5 and the rainwater utilization rate is 3%.

**Fig 9:**
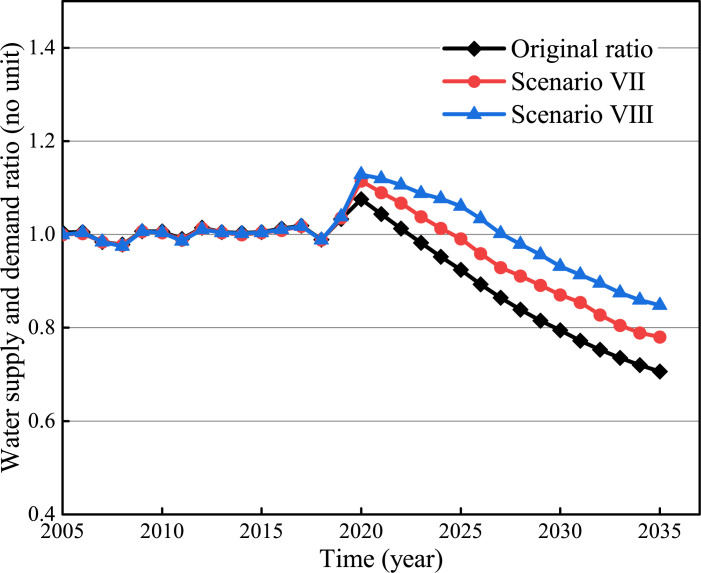
Optimization simulation results of unconventional water resources use.

Improvement in the utilization rate of wastewater can effectively increase the S: D ratio, shown in Fig 9. In 2025, the S: D ratio will increase from 0.92 to 0.99 (Scenario VII) and the corresponding utilization rate will increase by 38%. The S: D ratio will increase from 0.92 to 1.06 (Scenario VIII) and the utilization rate will increase by 51%. In 2035, the S: D ratio will increase from 0.71 to 0.78 (Scenario VII) and the corresponding utilization rate will increase by 45%. The S: D ratio will increase from 0.71 to 0.85 (Scenario VIII) and the utilization rate will increase by 61%. It is implied that unconventional water resources reuse and water-saving will need to be improved integrally to achieve a supplement to the total water supply [36, 49].

## Discussions

### Assessment of integrated planning schemes

To verify the optimization effect of all driving factors on the water resource supply and demand system, two groups of integrated optimization schemes referenced from national and local development plans were assessed, namely short-term planning and long-term planning.

Scenario IX: The S: D ratio reaches 1 in 2025. The values of the six parameters are set based on the minimum requirements stipulated in the national policies and local development plans [24].

Scenario X: The S: D ratio reaches 1 in 2035. The values of the six parameters are set based on the optimum requirements stipulated in the national policies and local development plans [24].

The values of the six parameters for the baseline scenario and the two designed scenarios are listed in Table 5.

**Table 5:** Integrated optimization scenarios.

Parameters	Baseline Scenario	Scenario IX	Scenario X
Domestic water demands per capita (m^3^/(person_*_year))	78.65	65.1	54.2
Tertiary industry water demand per capita (m^3^/(person_*_year))	26.33	15.7	7.7
Wastewater reuse rate (no unit)	0.3	0.4	0.6
Rainwater utilization rate (no unit)	0	1%	3%
Water consumption per ten thousand yuan of industrial value-added (m^3^)	22.3	22.25	20.81
Water demand per mu for farmland irrigation (m^3^/mu)	515	505	485

**Fig 10:**
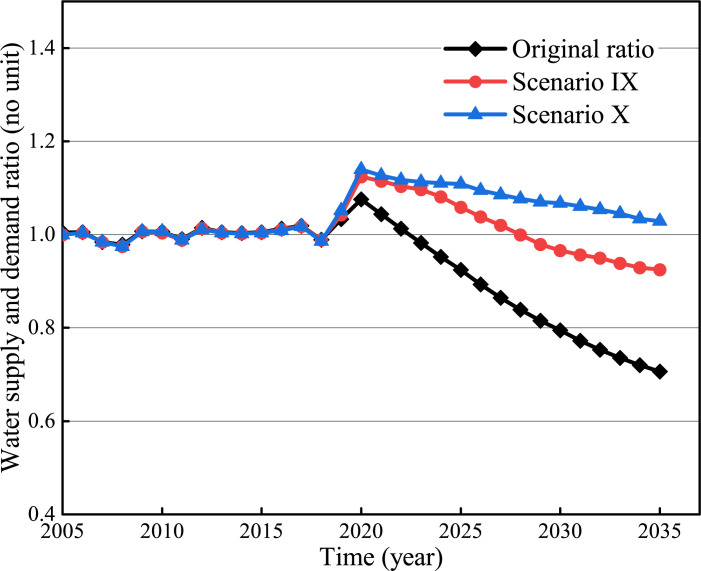
Integrated optimization simulation results.

Fig 10 shows that both the short-term and long-term optimization schemes can meet the sustainable planning of water resources. According to the short-term program (Scenario IX), the S:D ratio will increase from 0.92 to 1.06 in 2025. Additionally, the domestic water consumption, the tertiary industry water demand, and agricultural water use can decrease 27%, 24% and 4%, respectively. And the utilization of wastewater can be increased by 38%. The long-term program (Scenario X) indicates that S:D ratio will increase from 0.71 to 1.03 in 2035, by which the water consumption for household, tertiary industry and agricultural irrigation will be saved by 35%, 32% and 19%, respectively. And the unconventional water supply increased by 61%. It proves that bridging the gap between the water supply and demand means improving water supply not only controlling water demand [1].

### Simulation method of water resources supply and demand balance

The water resource supply and demand ratio can reveal whether the amount of available water resource in a district could afford its development requirements, representing a sustainable potential [50]. Its simulation and optimization involve a complicated systemic problem with the interaction of many subsystems [24, 51], but existing simulation methods often implement with a few influence factors and ignore its interaction, without considering the local development policies and plans. For example, Mirdashtvan et al. [13] only adopted adaptation scenarios to investigate the sustainable water supply and demand schemes. Li et al. [52] constructed a water resource accessibility index model considering five spatial factors to evaluate the water resource accessibility in Southwest China. Wang et al. [53] established a system dynamics model for the sustainable use of water resources in Chengde City, which involves the interaction among industrial, agricultural, and domestic water, but its scenario designs are limited on status quo type of simulated conditions. In this study, we combined variable interaction description and predictive simulation to analyze the water cycle system and proposed an optimal scheme for sustainable management of urban water resources by altering the driving factor values of water supply and water demand.

However, the water cycle simulation in this study still has some shortcomings. First, the water supply and demand simulation only considered the surface water, groundwater, and reclaimed water and ignored the climatic factors and water quality conditions [52]. Thus, in future research, the climate scenarios for water supply and water quality requirements for water demand should be integrated into water resource management simulation. Second, we only consider the volume of surface and underground water in the proposed model, ignoring its generation mechanism. At present, surface and groundwater models have been extensively studied. Combining different models at different scales can improve the understanding of actual water supply systems [54], and then combined models can be used to support water resources planning and management in a future study. Third, the achieved optimal scheme can meet the water supply and demand balance, but ignore the cost of saving water and unconventional water supply [55]. Therefore, the following research should include the economic cost in planning schemes selection [56].

### Water resources management for regional socio-economic development

The water resource supply and demand balance is an important basis for supporting socio-economic development. The policies and planning on the local blueprint were integrated to simulate the water resource variations. Although prior studies pointed out that restricting water demands [13, 57], improving water use for industry and wastewater reuse [24], increasing water saving [53], installing filtration plants to provide affordable water supply [58, 59] are all effective alternatives for achieving a water supply and demand balance, while simulation results in this study revealed that increasing unconventional water supply is more crucial than controlling water demand in improving water supply and demand balance. According to short-term and long-term optimization schemes, to guarantee balanced water supply and demand in the following 2025 and 2035, the domestic water consumption, the tertiary industry water demand, agricultural water use, and unconventional water supply should be consistently adjusted. This entails multiple administrative departments to coordinate and cooperate, such as the National Development and Reform Commission, Ministry of Housing and Urban-Rural Development, agriculture, education, Administration Bureau, etc. The progressive management regime and water-saving culture and experience of Chengdu lay the foundation for comprehensively promoting the construction of a water-saving society [60]. The municipal government takes measures such as differentiating the price of tap water and recycled water to induce the public to increase reclaimed water utilization [61]. Statistically, at least 132 million tons of reclaimed water was used to replenish water for river landscape, greenway, and wetland in Chengdu in 2020, and it is expected the reclaimed water utilization rate to be 50% by 2025 in Chengdu [62].

## Conclusions

A system dynamics model for water resource balance was developed to simulate the variation of water supply and demand in Chengdu, China. The sensitive factors were identified to drive the model from 2018 to 2035. Under the guidance of national and regional policies and development plans, this study further designed 8 scenarios to simulate the changes of the S: D ratio by changing key driving parameters.

The results show that the S:D ratio is sensitive to the industrial, agricultural, and domestic water demand, as well as the water consumption per ten thousand yuan of industrial value-added and rainwater utilization rate. Further, the S:D ratio will increase from 0.92 to 1.06 in 2025 via the short-term development program, from 0.71 to 1.03 in 2035 via the long-term program. It is thus suggested that improvement on the utilization rate of wastewater and utilization efficiency of water in service industries is essential to bridge the gap between the water supply and demand of megacities in China. In future policies, it is necessary to focus on improving pollutant treatment efficiency and wastewater reuse, and rainwater utilization rate. Therefore, it is imperative to coordinate the relationship between the economic cost of infrastructure and technology and water demand under regional hydroclimate and socioeconomic development in the future study [43]. Beyond the study region of Chengdu demonstrated in this paper, the constructed SD model can be applied to other megacities because of its generality in water supply and demand balance calculation and optimization by driver factors identification and scenario design procedures. The results can provide implications for city planners for sustainable water resources management by integrating local policies and planning.

## Supporting information

S1 Table Variable type of the SD model. (DOCX)

S2 Table The main equations of the SD model. (DOCX)
