# Supplementary material for: A scenario analysis-based optimal management of water resources supply and demand balance: A case study of Chengdu, China
Source: PLoS One. 2022 May 16;17(5):e0267920. doi: 10.1371/journal.pone.0267920 (PMC9109908; doi:10.1371/journal.pone.0267920)
Supplement: S1 Table — (DOCX) [file pone.0267920.s001.docx]

Parameters used in the system dynamics model to simulate the water resource supply and demand balance in Chengdu are shown in S1 Table (A represents auxiliary variable, R represents rate variable, L represents state variable and C is constant).

**S1 Table. Variable type of the SD model.**

| **Variable** | **Type** |
| --- | --- |
| Registered population growth rate | A |
| Non-registered population growth rate | A |
| Registered population growth | R |
| Non-registered population growth | R |
| Registered population | L |
| Non-registered population | L |
| Total amount of population | A |
| Growth rate of primary industry | A |
| Primary industry growth | R |
| Primary industry | L |
| Farmland irrigation area growth rate | A |
| Farmland irrigation area growth | R |
| Farmland irrigation area | L |
| Growth rate of forestry, animal husbandry and fishery | A |
| Forestry, animal husbandry and fishery growth | R |
| Gross product of forestry, animal husbandry and fishery | L |
| Growth rate of gross industrial production | A |
| Gross industrial production growth | R |
| Gross product of industry | L |
| Secondary industry growth | R |
| Secondary industry | L |
| Growth rate of secondary industry | A |
| Growth rate of tertiary industry | A |
| Tertiary industry growth | R |
| Tertiary industry | L |
| Ground water supply | C |
| Surface water supply | C |
| Rainwater utilization rate | A |
| Annual rainfall | C |
| Rainwater utilization | A |
| Wastewater reuse | A |
| Unconventional water supply | A |
| Wastewater reuse rate | A |
| Total amount of water supply | A |
| Domestic water demand | A |
| Domestic water demand per capita | A |
| Water demand per mu for farmland irrigation | A |
| Primary industry water demand | A |
| Forestry, animal husbandry and fishery water demand | A |
| Forestry, animal husbandry and fishery water consumption of ten thousand yuan | A |
| Industrial water consumption | A |
| water consumption per ten thousand yuan of industrial value-added | A |
| Urban ecological water demand | A |
| Tertiary industry water demand | A |
| Tertiary industry water demand per capita | A |
| Road cleaning and rivers and lakes ecological water supply | C |
| Green land water demand | A |
| Water consumption per unit green area | C |
| Green land area per capita | A |
| Green land area | A |
| Domestic wastewater discharge | A |
| Domestic sewage discharge coefficient | C |
| Industrial wastewater discharge | A |
| Industrial wastewater discharge coefficient | C |
| Total amount of wastewater | A |
