# Supplementary material for: A scenario analysis-based optimal management of water resources supply and demand balance: A case study of Chengdu, China
Source: PLoS One. 2022 May 16;17(5):e0267920. doi: 10.1371/journal.pone.0267920 (PMC9109908; doi:10.1371/journal.pone.0267920)
Supplement: S2 Table — (DOCX) [file pone.0267920.s002.docx]

**S2 Table. The main equations of the SD model.**

| No. | Type | Variables | Equations | Unit |
| --- | --- | --- | --- | --- |
| 1 | State equation | Registered population | =INTEG (registered population growth, 1082) | 10^4^ people |
| 2 |  | Non-registered population | =INTEG (non-registered population growth, 225.56) | 10^4^ people |
| 3 |  | Gross industrial production | =INTEG (gross industrial production growth, 757.3) | 10^8^ yuan |
| 4 |  | Primary industry | =INTEG (primary industry growth, 182.3) | 10^8^ yuan |
| 5 |  | Secondary industry | =INTEG (secondary industry growth, 1007.7) | 10^8^ yuan |
| 6 |  | Tertiary industry | =INTEG (tertiary industry growth, 1181) | 10^8^ yuan |
| 7 |  | Gross product of forestry, animal husbandry and fishery | =INTEG (forestry, animal husbandry and fishery growth, 149.72) | 10^8^ yuan |
| 8 | Rate equation | Registered population growth | =registered population* registered population growth rate | 10^4^ people |
| 9 |  | Non-registered population growth | =non-registered population*non-registered population growth rate | 10^4^ people |
| 10 |  | Forestry, animal husbandry and fishery growth | = Gross product of forestry, animal husbandry and fishery * growth rate of forestry, animal husbandry and fishery | 10^8^ yuan |
| 11 |  | Growth rate of forestry, animal husbandry and fishery | =With lookup (time [(2005,0)-(2035,10)],(2005,0.0757),(2006,0.2905),(2007,0.1761),(2008,0.1513),(2009,0.0675),(2010,0.1967),(2011,0.0128),(2012,0.0324),(2013,0.01382),(2014,-0.5164),(2015,0.4509),(2016,0.023),(2017,-0.14498),(2018,0.04)) | Dmnl |
| 12 |  | Primary industry growth | =primary industry  *growth rate of primary industry | 10^8^ yuan |
| 13 |  | Growth rate of primary industry | =With lookup (time [(2005,0)-(2035,0.5)], (2005,0.0851), (2006,0.0702), (2007,0.2071), (2008,0.1473), (2009,-0.0081), (2010,0.0638), (2011,0.148), (2012,0.0636), (2013,0.0147), (2014,0.011), (2015,0.0451), (2016,0.2728), (2017,0.0547), (2018,0.0439)) | Dmnl |
| 14 |  | Secondary industry growth | =secondary industry*growth rate of secondary industry | 10^8^ yuan |
| 15 |  | Growth rate of secondary industry | =With lookup (time [(2005,0)-(2035,30)], (2005,-0.01), (2006,0.2023), (2007,0.2413), (2008,0.2079), (2009,0.1019), (2010,0.2393), (2011,0.2672), (2012,0.1978), (2013,0.1104), (2014,0.0782), (2015,0.0477), (2016,0.1013), (2017,0.153), (2018,0.0864)) | Dmnl |
| 16 |  | Tertiary industry growth | =tertiary industry*growth rate of tertiary industry | 10^8^ yuan |
| 17 |  | Growth rate of tertiary industry | =With lookup (time [(2005,0)-(2035,0.7)], (2005,0.1861), (2006,0.1378), (2007,0.1795), (2008,0.1447), (2009,0.2308), (2010,0.2473), (2011,0.2492), (2012,0.1569), (2013,0.1364), (2014,0.1348), (2015,0.0989), (2016,0.1382), (2017,0.1381), (2018,0.1237)) | Dmnl |
| 18 |  | Gross industrial production growth | =gross industrial production * growth rate of gross industrial production | 10^8^ yuan |
| 19 |  | Growth rate of gross industrial production | =With lookup (time [(2005,0)-(2035,30)], (2005,-0.0410), (2006,0.2197), (2007,0.2703), (2008,0.2608), (2009,0.1253), (2010,0.2391), (2011,0.2657), (2012,0.1579), (2013,0.1019), (2014,0.0937), (2015,0.0521), (2016,0.1042), (2017,0.1648), (2018,0.0857)) | Dmnl |
| 20 | Auxiliary equation | Total water demand | =urban ecological water demand + domestic water demand + primary industry water demand + secondary industry water demand + tertiary industry water demand | 10^8^ m^3^ |
| 21 |  | Total GDP | =primary industry + secondary industry + tertiary industry | 10^8^ yuan |
| 22 |  | Total amount of population | =registered population + non-registered population | 10^4^ people |
| 23 |  | water supply and demand ratio | =total water demand / total amount of water supply | Dmnl |
| 24 |  | Total amount of water supply | =ground water supply + surface water supply + unconventional water supply | 10^8^ m^3^ |
| 25 |  | Domestic water demand | =domestic water demand per capita*total amount of population / 10000 | 10^8^ m^3^ |
| 26 |  | Industrial water consumption | =water consumption of ten thousand yuan of industrial value-added*gross product of industry / 10000 | 10^8^ m^3^ |
| 27 |  | Agricultural irrigation water demand | =water demand of per mu for agricultural irrigation*agricultural irrigation area / 10000 | 10^8^ m^3^ |
| 28 |  | Domestic wastewater discharge | =domestic water demand*domestic sewage discharge coefficient | 10^8^ m^3^ |
| 29 |  | Industrial wastewater discharge | =industrial wastewater discharge coefficient*industrial water consumption | 10^8^ m^3^ |
| 30 |  | Wastewater reuse | =wastewater reuse rate*total amount of wastewater | 10^8^ m^3^ |
| 31 |  | Green land area | =green land area per capita*total amount of population | 10^4^ m^2^ |
| 32 |  | Green land water demand | =water consumption per unit green area*green land area/10000 | 10^8^ m^3^ |
| 33 |  | Rainwater utilization | =rainwater utilization rate*annual rainfall | 10^8^ m^3^ |
| 34 |  | Total amount of wastewater | =domestic wastewater discharge + industrial wastewater discharge | 10^8^ m^3^ |
